# LC-MS/MS and LC-PDA Methods for Robust Determination of Glycerol Phenylbutyrate in Biological Fluids and High-Resolution Mass Spectrometric Identification of Forced Degradation Product and Its Whiteness

**DOI:** 10.1021/acsomega.5c00569

**Published:** 2025-04-25

**Authors:** Serkan Levent, Abeer Elriş, Hazal Avcı, Ülfet Erdoğan Uzunoğlu, Saniye Özcan, Nafiz Öncü Can

**Affiliations:** 1 Department of Analytical Chemistry, Faculty of Pharmacy, Anadolu University, Eskisehir 26470, Turkey; 2 Central Analysis Laboratory (MERLAB), Faculty of Pharmacy, Anadolu University, Eskisehir 26470, Turkey; 3 Department of Chemistry and Biochemistry, Clarkson University, Potsdam, New York 13699-5810, United States

## Abstract

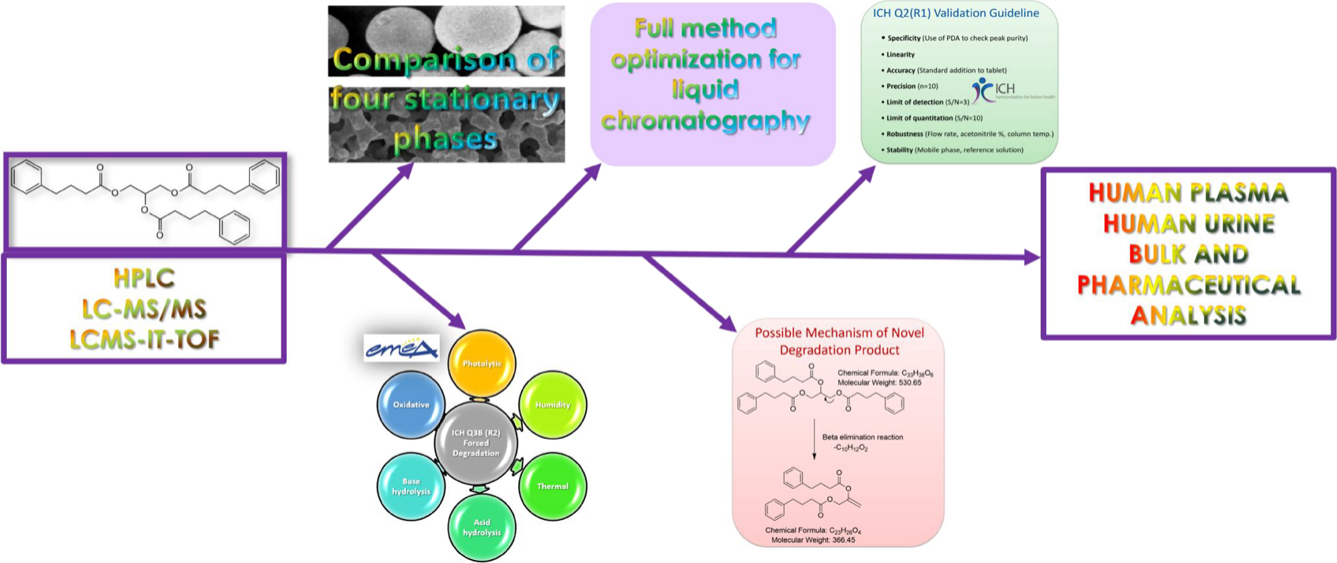

In 2013, the FDA approved glycerol phenylbutyrate to treat urea cycle disorders in people who could not go through 2 months of protein restriction and/or amino acid supplementation. The paper suggested a simple, quick, and eco-friendly liquid chromatographic method to analyze glycerol phenylbutyrate in Ravicti, pharmaceutical formulation, bulk, human urine, and plasma. Also, a novel degradation product was characterized by applying severe degradation conditions, according to the ICH Q1A(R2) guideline. The liquid chromatography conditions were 0.5 mL/min flow rate and 1 mM ammonium acetate buffer:acetonitrile (25:75; *v*/*v*) (≈ pH 5.30). The system backpressure was 67 bar. A core–shell particle column (Ascentis Express F5 2.7 μm, 100 × 4.6 mm i.d.) from Supelco was used for separation. The method was fully validated according to the ICH Q2(R1) guideline. The method linearities for bulk and pharmaceutical analysis were 1.40–55.84 ng/mL for LC-PDA and 2.79–111.68 μg/mL for LC-MS/MS. Indeed, for the plasma sample, the lowest recovery was LC-PDA and LC-MS/MS achieving 94.27 and 98.20%, respectively. Moreover, in forced degradation experiments, the active substance was unstable in acid, alkali, and oxide conditions, and an elimination reaction forms the novel degradation product. Lastly, the method was evaluated to have excellent whiteness, efficiency, and practicality, making it suitable for application in all analytical method development laboratories.

## 1 Introduction

Urea cycle disorders (UCDs) are metabolic birth defects that happen when any of the six enzymes that the liver needs to change urea in the kidneys do not work right. In this situation, the liver gets rid of ammonia from the blood. There are two types: not having enough of certain enzymes that turn fats or carbohydrates into energy and not having enough of certain enzymes that break down amino acids or other metabolites. Both can build up and become harmful if not treated.^1^ The clinical symptoms of UCDs in adults are diverse. It varies from life-threatening poisoning in neonates to asymptomatic conditions in adults. Early diagnosis is essential to decreasing death and preventing irreparable neurological injury.^2^ The reversibility of the harmful effects of hyperammonemia depends on the duration of exposure, its severity, and the stage of brain development. Hyperammonemia in the newborn brain induces edema due to astrocytic swelling. The frequency and size of the subsequent swelling are important factors in determining how bad neurological problems in the central nervous system are, such as seizures, coma, and problems with thinking or moving.^3^

Glycerol phenylbutyrate (GPB) was approved by the FDA in 2013 for the treatment of UCDs in people who could not handle 2 months of protein restriction and/or amino acid supplementation. The nitrogen conjugation of drugs that is unknown used in young patients and their pancreatic exocrine function may not be fully developed yet.^4^ Three phenyl butyric acid molecules, linked to glycerol through an ester bond, compose GPB. It is a colorless, odorless, and almost tasteless liquid that works by providing a different way to get rid of ammonia and waste nitrogen through the urine as phenylacetyl glutamine.^5^

GPB is a triester formed from three phenylbutyrate parts and glycerol. GPB serves as a prodrug for phenylacetate. When taken by mouth, it breaks down right away in the small intestine, creating the prodrug phenylbutyrate. It is then absorbed through the small intestines. After that, phenylbutyrate goes through mitochondrial beta-oxidation in the liver, which makes phenylacetic acid, which is an active compound. Phenylacetic acid conjugates with glutamine to produce phenylacetylglutamine in the liver and kidney. Phenylacetylglutamine is a urea substitute that provides an alternative mechanism for nitrogen excretion. It eliminates excess nitrogen from urine, thereby lowering ammonia levels.^6,7^ The determination of the GPB dosage takes into account several parameters, including body surface area, daily protein consumption, the urea cycle enzymatic function, initial dosage in phenylbutyrate-naive patients 4.5 to 11.2 mL/m^2^/day, and the maximum total daily dosage of 17.5 mL (19 g)^8,9^

Under the brand name Ravicti, GPB stands as the most expensive medication in the United States, according to 2023 data. GPB had 7 years of market privilege for each determined indication, specifically from February 2013 to February 2020 for adult and pediatric patients aged 2 years and older, and from April 2017 to April 2024 for pediatric patients aged 2 months to 2 years.^10^ GPB is a chemical compound with the chemical name 2,3-bis(4-phenylbutanal oxy)propyl 4-phenylbutanoate; the molecular formula is C_33_H_38_O_6_, and its molecular weight is 530.6 g/mol.

To the best of our knowledge, there are currently no published data on the analysis of glycerol phenylbutyrate using liquid chromatography. This is the first study of the analysis of GPB for pharmaceutical or biological fluids.

The International Conference on Harmonization (ICH) advocates for the full validation and stability-indicating nature of analytical tests for stability samples. Moreover, guidelines recommend that forced degradation studies be performed to establish the stability behaviors of the drug molecule. For instance, the degradation pathways and the identification of degradation products enhance the acceptability of the developed analytical method.^11^

The purpose of this work is to offer an easy, low-cost, and accurate liquid chromatographic method that could be used for analyzing GBP in bulk, pharmaceutical formulation, human urine, and plasma. The manuscript includes two distinct, fully validated applications applicable to both photo diode array detector (PDA) and tandem mass detectors. Furthermore, we performed a PDA detection approach in the presence of its degradation products, which led to the identification of a novel degradation product (DP). The novel degradation product was characterized using a high-performance liquid chromatograph with an ion-trap and time-of-flight mass spectrometer (LC-MS-IT-TOF) and suggested a possible formed mechanism. Additionally, the developed approaches were assessed and compared according to the white analytical chemistry (WAC) approach.

## 2 Experimental Section

### 2.1 Reagents and Chemicals

LC-MS-grade acetonitrile was procured from J. T. Baker (USA), LC-MS-grade ammonium acetate was purchased from Fisher Chemicals (USA), and analytical reagent-grade sodium hydroxide, hydrogen peroxide, hydrochloric acid, and trifluoroacetic acid (TFA) were purchased from Sigma-Aldrich (USA). GPB reference standard was obtained from Molekula (Germany). Ultra-standard water obtained from the Milli-Q water unit (USA) was utilized throughout the studies.

### 2.2 Instrumentation

In the experiment, two different liquid chromatography systems from Shimadzu (Japan) were used. An LC-MS-8040 with a Nexera XR-series modular LC for LC-MS/MS and LC-PDA experiments (instrument 1) was equipped with a DGU-20A3R model degassing unit, an LC-20AD binary gradient pump, a SIL-20AC cooling autosampler, a CTO-10ASVP column oven, a CBM-20A communications bus module, and an SPD-M20A PDA detector. The computer connected to the LC system had a Windows 10 software system, and the LCSolutions 1.11 SP1 data analysis program was used for system control and obtaining chromatograms.

For interlaboratory comparison, Shimadzu UFLC (instrument #2) was utilized. This tool consists of a DGU-20A3 degasser unit, two LC-20AD gradient pumps, an SIL-20ACHT autosampler, a CTO-10ASVP column oven, a CBM-20A communications bus module, and an SPD-M20A PDA detector. It is operated using Windows 10 and LCSolutions 5.81 software for data and system control.

We conducted qualitative high-resolution mass spectrometric determinations using an LC-MS-IT-TOF Shimadzu (Japan), a technique based on the principle of utilizing an ion-trap time-of-flight spectrometer. It has a DGU-20A3R 3-line degassing unit, two LC-20AD dual gradient pumps, a SIL-20A HT autosampler, a CTO-10ASVP column oven, SPDD-M20A PDA detectors, and a CBM-20A communications unit. The LC system utilized a computer-running Windows XP and Shimadzu LC LabSolutions 3.43 SP1 data analysis program.

The utilized stationary phase was a core–shell particle column (Ascentis Express F5 2.7 μm, 100 × 4.6 mm inner diameter) from Supelco (Sigma-Aldrich, USA). A Mettler Toledo (Switzerland) SevenMulti model pH meter, an XSE 105 series dual range model analytical balance, a Heidolph (Germany) Reax Top model vortex, and a Bandelin Sonorex (Germany) RK 100 H model ultrasonic bath were used in experimental part.

### 2.3 Instrumental Parameter and Chromatographic Conditions

The chromatographic separation was conducted using LC, specifically employing a Supelco Ascentis Express F5 column (2.7 μm, 100 mm × 4.6 mm). The mobile phase consisted of 1.0 mM ammonium acetate buffer and acetonitrile in a 25:75 (*v*/*v*) ratio. The flow rate was maintained at 0.5 mL/min, with an injection volume of 1.0 μL. The column temperature was controlled at 40.0 ± 0.1 °C, while the autosampler was held at 15 ± 0.1 °C. The buffer solution was obtained by dissolving 77.08 mg of ammonium acetate in 1 L of water and sonicated for 5 min, and then the pH of the mobile phase was measured and adjusted to ≈5.30 if needed. Prior to analysis, all mobile phase solutions were filtered using a 0.22 μm PVDF filter.

The MS detector was monitored in the mass range from *m*/*z* 100 to *m*/*z* 800, utilizing ESI+ and ESI– with MRM. Other conditions were optimized: nebulizing gas (N_2_) flow rate at 3.0 L/min; drying gas (N_2_) flow rate at 15 L/min; collision gas as Ar, heat block temperature set to 450 °C; CDL temperature at 250 °C; and dwell time of 100 ms.

The PDA detector was configured for all instruments at the wavelength 200 nm, corresponding to the maximum absorbance of GPB. Additionally, the spectra were explored at a data-sampling frequency of 1.5625 Hz and within the wavelength range of 190 to 380 nm.

An LC-MS-IT-TOF mass spectrometer with an ESI interface was utilized for high-resolution mass spectrometry analysis. The requirements for analysis were defined as follows: nebulizing gas flow 1.5 L/min; drying gas pressure 200 kPa; high-voltage probe potential −3.5 kV; heat block temperature 200 °C; CDL temperature 200 °C. The CID parameters consist of 50% for the collision gas, 50% for CID energy, and argon gas as the CID medium. Additionally, the detector voltage of the TOF was maintained at 1.6 kV, and the IT-TOF system underwent calibration using a sodium trifluoroacetate solution.

### 2.4 Preparation of the Standard and Sample Solutions

A stock solution of 1 mg/mL GPB was prepared in acetonitrile because of the solubility of GPB. Further dilutions for the LC-MS/MS and LC-PDA methodologies of low-concentration solutions were made with acetonitrile. The stock solution was stored at −20 °C.

10.0 mg of GBP solid was carefully measured on a scale and placed in a 5 mL flask and then filled up with acetonitrile. Then, to prepare 5 mL of 1 N HCl solution in water from 36% (*w*/*w*) stock HCl solution, 413 μL of stock HCl solution was taken and transferred to a flask containing some water, and the volume was completed with water. For basic forced degradation studies, 200 mg of NaOH solid was weighed and transferred to a flask containing some water, and the volume was completed with water. For oxidative forced degradation studies, 1000 μL of 30% (*w*/*w*) stock H_2_O_2_ solution was taken and transferred to a flask containing some water, and the volume was completed with water. In the forced degradation experiments, 500 μL of stock GPB solution (2 mg/mL) was taken into a 1.5 mL glass vial and 500 μL of forced degradation solution (acid, base, or oxidative) was added. For the UV-light and heat forced degradation solution, 500 μL of the stock GPB solution was taken, and 500 μL of water was added. While the blank forced degradation solutions were prepared, 500 μL of the relevant forced degradation solution was added with 500 μL of acetonitrile instead of the GPB stock solution. So, the final concentration of the forced degradation solution was 1 mg/mL GPB and acid and base solutions 0.5 N; the oxidative condition was 3% H_2_O_2_. We vortexed all forced degradation solutions for 30 s and then immediately injected them into the LC system for a duration of 0 min. Next, a temperature of 60 °C was used, and the chromatograms and spectra from the blank solutions and earlier measurements were compared and analyzed.

For human urine samples, 1 mL of GPB standard solution was spiked to 9 mL of human urine and vortexed for 3 min. For blank human urine samples, 1 mL of acetonitrile was added to 9 mL of human urine and vortexed for 3 min. After they were centrifuged at 4000 rpm for 10 min and 3 mL, their supernatants were filtered through 0.22 μm PVDF. From each supernatant, 2 mL of solution was diluted to 10 mL with water and injected into the LC system.

For human plasma samples, 100 μL of GPB standard solution was spiked to 1 mL of plasma and vortexed for 3 min. For blank plasma samples, 100 μL of acetonitrile was added to 1 mL of plasma and vortexed for 3 min. Afterward, 2 mL of acetonitrile was added, vortexed for 3 min, and centrifuged at 4000 rpm for 10 min, separately. The supernatants were filtered through 0.22 μm PVDF into the LC system.

### 2.5 Forced Degradation Studies

The ICH (Q2)R1 guideline was offered to forced degradation studies, which included photolysis, oxidation, dry heat, and hydrolysis in various media (acidic, basic, and neutral).^11^ The study used degradation solutions consisting of aqueous hydrochloric acid at 0.5 N, aqueous sodium hydroxide at 0.5 N, and H_2_O_2_ at 3% (*w*/*w*). To achieve the degradation procedure, standard GPB was prepared in acetonitrile and all degradation solutions were treated with standard GPB solution. The final GBP concentration was set at 1 mg/mL in all solutions. Moreover, other degradation conditions included exposure to UV light for 12 h, 75% humidity for 1 h, and 1 h in 10 °C increments at a constant temperature of 60 °C. The general outline of the degradation studies is given in Figure S1.

### 2.6 Method Validation Protocol

#### 2.6.1 Selectivity

According to ICH guidelines, specificity and selectivity refer to the ability to detect the target analyte(s) in the presence of related substances using the proposed method. To prove specificity and selectivity, you must show the peak purity that does not match up with any interferences at the same time as the analyte(s)’s retention time. Throughout the method development stage, we demonstrated the absence of any suspicious or unexpected peaks in the chromatograms or spectra with stability, indicating analysis. Also, the peak purity spectra and purity results were calculated.

#### 2.6.2 System Suitability

In addition, the validation parameter system suitability tests (SSTs) were also demonstrated for the chromatographic performance of the LC instrumentation. The presented system suitability parameters are as follows: number of theoretical plates (*N*), tailing factor (*T*), resolution (Rs), and capacity factor (*k*).

#### 2.6.3 Linearity

For the LC-MS/MS and LC-PDA instruments, the linearity concentrations were different and nine concentration points were used to make solutions for each. Linearity was determined in the ranges 1.40–55.84 ng/mL for LC-MS/MS and 2.79–111.68 μg/mL, respectively. For both methods, a linear regression model was utilized. The slope, intercept, correlation coefficient, and confidence intervals are calculated at the 95% confidence level.

#### 2.6.4 Precision

Recovery studies determined the accuracy of the method. The recovery experiments were performed in plasma and urine medium. Blank plasma and urine samples were spiked with the concentration of the linearity range corresponding to the LOQ, 80, 100, and 120% for each method. For each level, three parallel runs occurred, and results were explained as mean recovery with standard deviation and confidence limits at a 95% confidence level and RSD%. Moreover, one-way ANOVA was calculated for the significant statistical comparisons. In addition, the method was tested on interlaboratory comparison in order to ensure the transferability and variation between laboratories.

#### 2.6.5 Accuracy

Recovery studies determined the accuracy of the method. The recovery experiments were performed in plasma and urine medium. Blank plasma and urine samples were spiked with the concentration of the linearity range corresponding to LOQ, 80, 100, and 120% for each method. For each level, three parallel runs occurred, and results were explained as mean recovery with standard deviation and confidence limits at a 95% confidence level and RSD%.

#### 2.6.6 Limits of Quantitation and Detection

The ICH guideline recommends various methods to find the LOD and LOQ values of the developed methods. This study utilized the device’s signal-to-noise (S/N) ratio to determine the LOD and LOQ values. In order to determine LOD, the concentration, which is S/N, equal to 3, was prepared and analyzed six times consecutively. Similarly, we prepared and analyzed the CPB solution, whose S/N was 10, to determine the LOQ.

#### 2.6.7 Stability

The GBP stock solution was diluted to correspond to 100% concentration of linearity, and the solution was analyzed periodically. The time periods were as follows: 6, 12, 18, 24, and 48 h, at −20 °C for 3 weeks and three freeze–thaw cycles. The analysis for each time period was conducted three times.

#### 2.6.8 Robustness

The robustness of a method entails evaluating and comparing optimal outcomes with respect to key response parameters, such as theoretical plate number, retention time, resolution, and tailing factor, while systematically altering optimal method conditions and remeasuring. In this study, the flow rate, mobile phase acetonitrile percentage, and column temperature were deliberately changed, and the theoretical plate number and retention time were measured as responses.

## 3 Results and Discussion

### 3.1 Chromatographic Studies

The method optimization stage was started with preliminary experiments in the existence of GPB and DP(s). To achieve good separation, SST parameters, and the shortest analysis time, various mobile phase contents, mobile phase pH, and column types were tried. In the process, it has occurred to maintain one variable constant, alter the other, and track the responses. The GPB logP value is 6.5, making it nearly insoluble in water but soluble in organic solvents, such as dimethyl sulfoxide or acetonitrile. We tried four types of columns as stationary phases, and each column exhibited highly diverse responses. These are the first groups of columns: Supelco Ascentis Express Phenyl Hexyl 100 × 4.6 mm, 2.7 μm, and Supelco Ascentis Express F5 100 × 4.6 mm, 2.7 μm. Such columns mentioned above consist of particulate silica. These columns, Chromolith HR RP-18e 100 × 4.6 mm and Chromolith Performance RP-18e 100 × 2 mm, are different from the others that were used for optimization tests because they are made of C18-bonded second-generation monolithic silica. We observed the highest tailing factor and unexpected baseline noise when using Chromolith HR RP-18e 100 × 4.6 mm. The SST parameters for the Chromolith HR RP-18e 100 × 4.6 mm column were acceptable, but the longest retention time was unacceptable for WAC rules. The silica particle columns had many advantages compared to the C18 column in the experiments. Silica particle columns are especially good at separating compounds whose chemical and physical properties are very similar. This is especially useful for separating impurities or analyzing DPs.^12^ In relation to this, the responses of the columns packed with silica particles were superior. In the chromatograms obtained from Supelco Ascentis Express, phenylhexyl had a low tailing factor and retention time, and NTP was high. Supelco Ascentis Express F5 performed better than other columns for each SST parameter due to its π–π interactions between the electron-deficient fluorinated phenyl group and electron-rich aromatic eluents, with PFP columns providing effective separation for aromatic or heterocyclic compounds. Consequently, this column achieved the best SSTs, such as the highest NTP and the lowest tailing factor. For this reason, pentafluorophenylpropyl (F5) functional groups were utilized as the stationary phase, and obtained results are summarized in Figure S2 for every mobile phase type.

**Figure 1:**
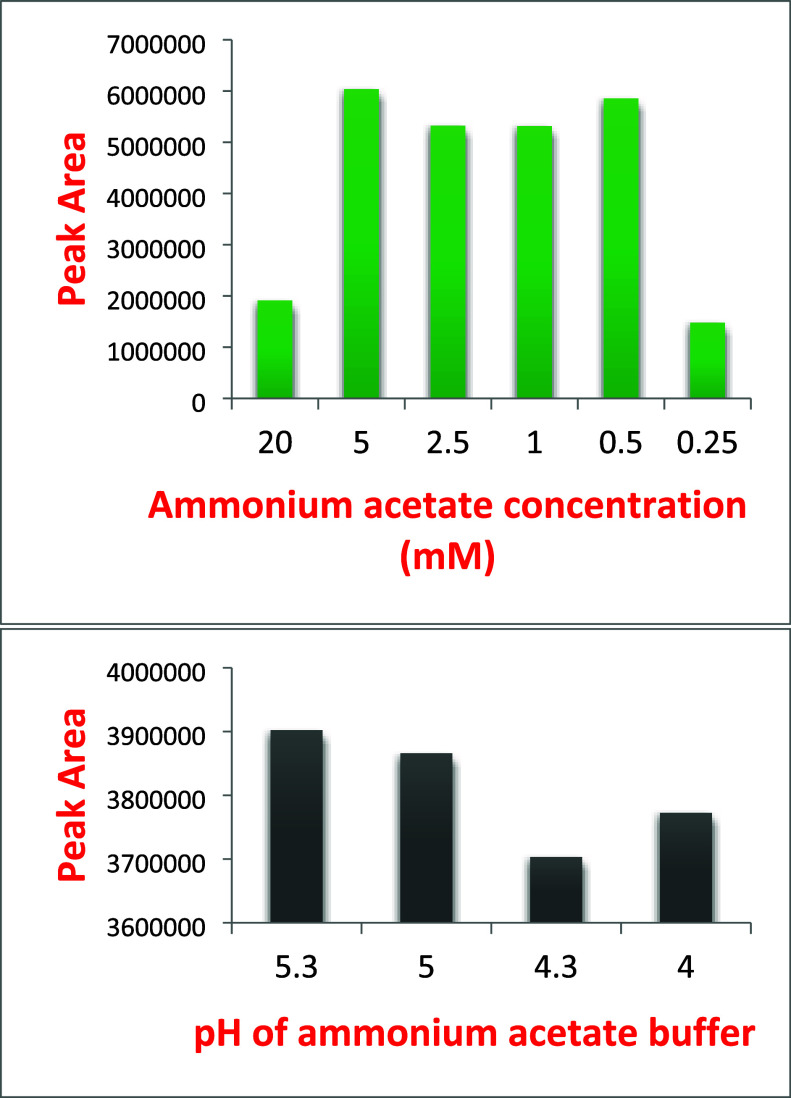
Effect of mobile phase additive and pH on the peak area of GPB.

When the developed method had to be adapted to high-resolution mass spectrometric detection, many important factors were taken into account in the selection of the mobile phase. The phenomena included volatility of the mobile phase additive, concentration of the salt buffer, and flow rate. During the method optimization process, both methanol and acetonitrile were tried as mobile phase organic components. Due to the low elution time, methanol was not preferred. Because of its weakly basic GPB p*K*_a_ value of −6.6, it naturally ionizes at lower pH levels. So, ammonium acetate was chosen as a mobile phase additive, and different concentrations and pH values of the mobile phase. Good peak morphology and separation were achieved. The best peak shape was obtained with 1 mM, pH 5.3, ammonium acetate buffer. Finally, the mobile phase is made up of acetonitrile and ammonium acetate buffer (1 mM each) mixed at 25:75 (*v*/*v*) (pH 5.30), and it flows at a rate of 0.5 mL/min. The mobile phase buffer concentration and pH relation with peak area are given in Figure 1. After figuring out the column and mobile phase composition, the following other factors led to the best separation for GPB when DP(s) was present: 40.0 °C column temperature and 1 μL injection volume. To sum up with the chromatographic separation, conditions were optimized in the presence of DPs of GPB. The maximum intensity of DP was recorded in an oxidative medium. Additionally, under acidic conditions, DP was detected. The observation and corresponding degradation solutions formed the primary basis for all optimization studies. The total analysis time was 6 min, which was sufficient to elute and separate DP and GPB. Maximum absorbance of GPB was detected at 200 nm. The SST was evaluated according to the USP. All values are in the range of recommendation. The results are listed in Table 1.

**Table 1:** System Suitability Tests and Linearity of the Method

**system suitability and linearity parameter**	**acceptance criteria**	**LC-MS/MS**	**LC-PDA**
injection precision for area (*n* = 6)	RSD ≤ 1%	0.299	0.126
injection precision for retention time (min)	RSD ≤ 1%	0.142	0.051
RSD (%5) of area response factor	RSD ≤ 1%	0.842	0.808
USP tailing (*T*) for GPB peak	*T* ≤ 2	1.113	0.951
theoretical plates (*N*) GPB peak	*N* ≥ 2000	7054	9088
linearity range		1.40–55.84 ng/mL	2.79–111.68 μg/mL
slope ± SE (intraday, *n*= 9)		3897.9 ± 57.6	6741.5 ± 96.4
intercept ± SE (intraday, *n*= 9)		1549.7 ± 1666.0	3310.3 ± 5576.0
regression coefficient (intraday, *n* = 9)		0.9985	0.9986
limit of detection		0.105 ng/mL	0.689 μg/mL
limit of quantification		1.149 ng/mL	0.957 μg/mL
slope ± SE (interday, *n* = 27)		3871.9 ± 70.1	6830.5 ± 109.8
intercept ± SE (interday, *n* = 27)		1576.6 ± 2027.6	–7479.5 ± 6351.9
regression coefficient (interday, *n* = 27)		0.9977	0.9982
ANOVA		*F* = (2,24) = 0.00055	*F* = (2,24) = 0.00061
		*P* = 0.9995 (*P* > 0.05)	*P* = 0.9994 (*P* > 0.05)

### 3.2 LC-MS/MS Studies

**Figure 2:**
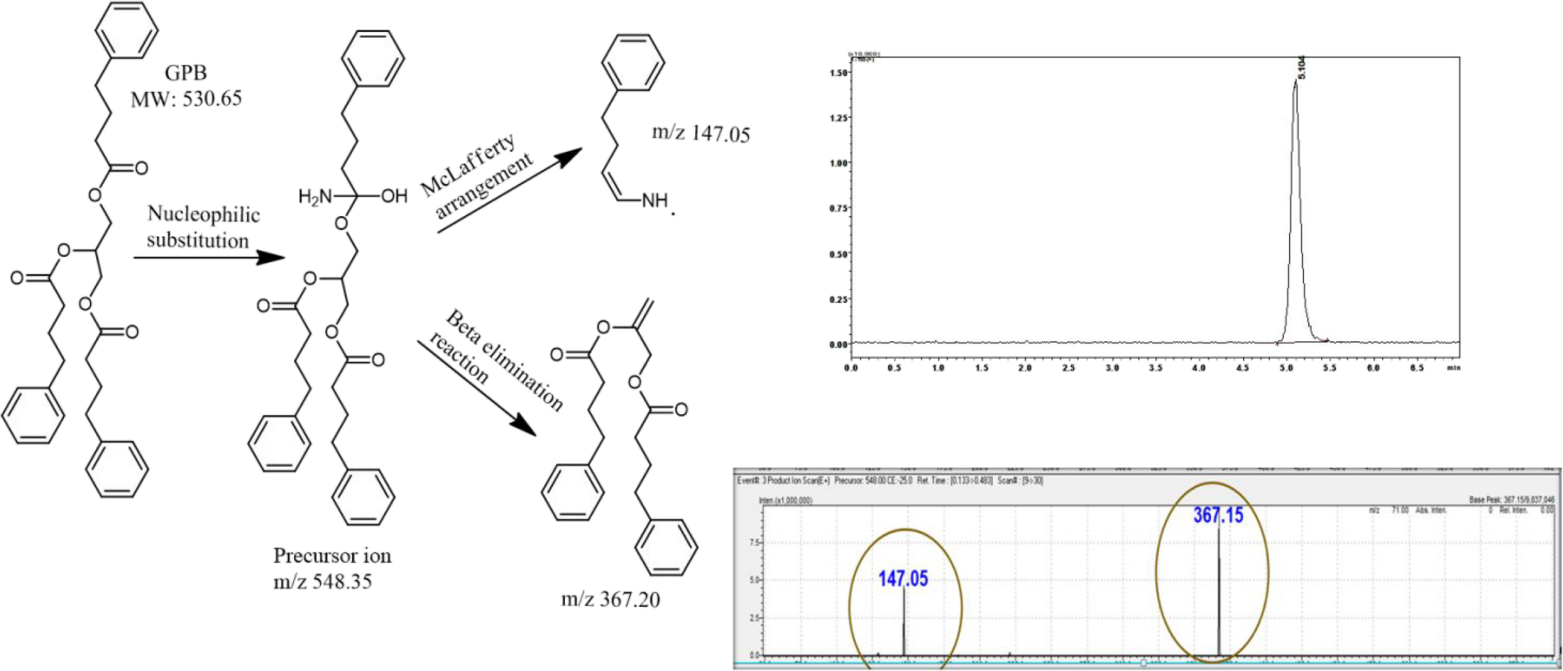
Total ion chromatogram and MS/MS fragmentation of GPB (27.92 ng/mL).

For low detection and quantification limits, the LC-MS/MS method was developed. The optimized method that was mentioned above employed for HPLC was utilized without substituting the buffer. The phenomena were improved for ionization in the source and made it easier to find DPs, even at low concentrations. The nebulizing gas flow, drying gas flow, drying gas temperature, and spray voltage were all changed. For the method, a concentrated solution was made, and a preliminary study was carried out to find the best type of ionization for the GPB. The best energy for the ions that come from the ESI+ MRM mode is shown in Table 2 and dwell time managed as 100 ms for each product ion. When mass spectrometry was set up, the precursor ion was split into two daughter ions with high-quantitation *m*/*z* ratios of 367.20 and 147.05 as given in the total ion chromatogram in Figure 2. The possible formation mechanism of precursor ion was nucleophilic substitution in ammonium acetate medium. So, amide could be formed. The daughter ion that has *m*/*z* ratio 147.05 could be formed for Mclafferty rearrangement. The other daughter ion that has *m*/*z* ratio 367.20 could be formed for beta elimination reaction. The proposed ionization mechanism is shown in Figure S3.

**Table 2:** ESI+ Mode MRM Conditions of GPB

**compound**	**precursor ion**	**product ion**	**Q1 Pre Bias (V)**	**CE(V)**	**Q3 Pre Bias (V)**
GPB	548.35	367.20	–34.0	–19.0	–25.0
		147.05	–20.0	–33.0	–26.0

### 3.3 LC-MS-IT-TOF Studies

The ICH Q3B (R2) guideline offers the identification and quantification of any impurities exceeding 0.1%.^13,14^ The literature has described the advantages of high-resolution mass spectrometry (HRMS) for impurity profiling, quality control, and surveillance. However, chromatography traditionally performs the assessment of impurities.^15^ HRMS was achieved with good resolution by enhancing sensitivity and/or scanning speed. HRMS studies provide us with the opportunity to see the exact isotopic distribution of an ion. This makes it easier to assign molecular formulas by comparing experimental data to theoretical isotopic distributions. When you use isotopic fine structures and unique molecular formula assignments with MS/MS experiments, it is easier to figure out the molecule structure. To sum up, HRMS is useful for studying drug formulation because it is very sensitive and could examine with both compounds and impurities at very low concentrations with fragmentation experiments.^16^

So, the researchers preferred a sensitive and detailed LC-MS-IT-TOF method to identify DP(s). The method development studies were carried out under the same conditions as for the MS/MS experiments. The mobile and stationary phases were switched, and the best conditions were set up.

### 3.4 Stability and Degradation Studies

The solutions prepared for the degradation experiment were originally maintained at room temperature. Then, all solutions were gradually heated to 60 °C to produce degradation substances. All solutions were analyzed using the LC-MS-IT-TOF to characterize possible DPs of GPB. The maximum heating period for all solutions was totally 3 h, but 1 h was sufficient to detect the DP. For the heat forced degradation behavior, GPB was treated at 60 °C directly. GPB was stable under the condition that no DPs were detected. Under room-temperature oxidatively forced degradation conditions, GPB exhibited stability. However, upon heating to 60 °C, the degradation occurred. GPB was treated to a 3% H_2_O_2_ solution at 60 °C, resulting in the detection of a novel molecule with a *m*/*z* of 367.1912. Furthermore, in acidic and basic media maintained with 0.5 N HCl and 0.5 N NaOH at room temperature, decomposition was not observed; however, when heated to 60 °C, the previously obtained DP reappeared but with less intensity than under oxidative conditions. According to its MS (ESI-IT-TOF) spectrum, it was identified using a novel DP with a molecular weight of 367.1912 g/mol, as shown. A novel DP was identified under oxidative thermal degradation conditions. Figures S4–S8 present overlay PDA chromatograms of blank and sample solutions obtained under each forced degradation condition from the LC-MS-IT-TOF instrument. The peak of the novel DP was determined as the retention time of approximately 1.35 min under all three forced degradation conditions. Although the decomposition rate was quite slow under experimental conditions, it could be detected even with a PDA detector after 1 h. Additionally, the mass balance results of GPB and its DPs are shown in Table S1 for 18 h.

Although there are many methods to calculate mass balance, since the qualitative determination of DPs is in the foreground here, it was calculated from peak areas.^17^ Here, the peak areas represent the sum of the total mass of the reference sample and the degraded sample. Since the DPs are unknown, their standards cannot be calculated. So, in studies of degradation, all peak areas that are present in the analysis chromatogram but not in the blank chromatogram and make up more than 0.10% of the total peak area were looked at. Percent impurities were calculated using peak areas. According to these experiments, the highest loss in the active substance at the end of 18 h was obtained under thermal oxidative forced degradation conditions and was calculated as 3.9%. In thermal acidic degradation conditions, the loss was calculated as 1.4%. The least amount of DP was produced under basic thermal basic conditions. In heat- and light-forced degradation conditions, no loss was observed in the mass balance of the active substance.

**Figure 3:**
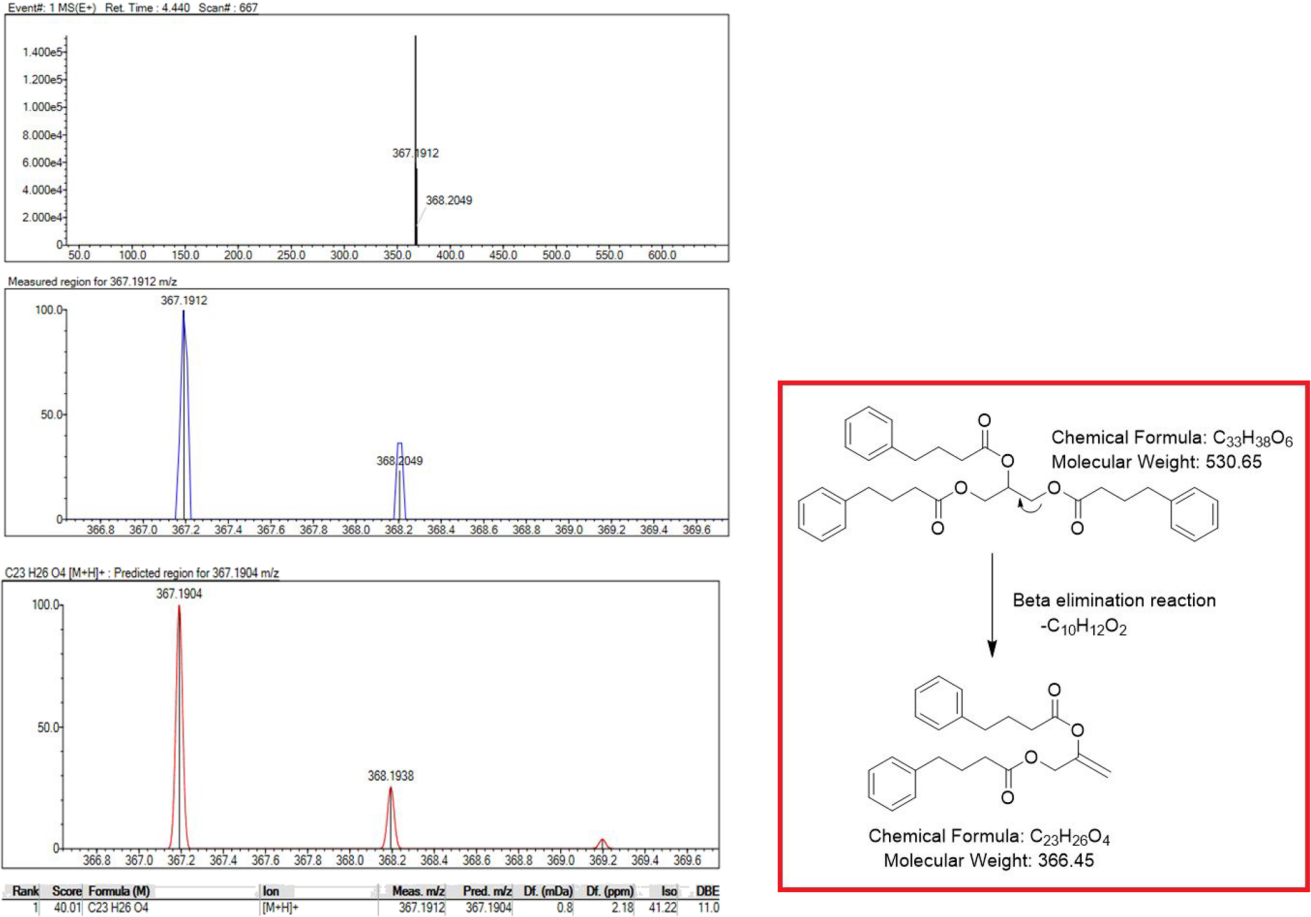
Characterization of the degradation product of GPB and its possible mechanisms.

Figure 3 presents the acquired MS spectrum along with a potential production mechanism. The removal of an ester group from the main GPB molecule under oxidative stress conditions results in the formation of a novel DP. A possible formation mechanism of the novel DP was that the unpaired electrons on the oxygen engage with the H+ ions in an oxidative medium, resulting in cleavage of the C–O bond and formation of a new pi (π–π) bond. The new DP is revealed by the beta elimination reaction. So, the molecule is unstable and not resistant to forced oxidative degradation conditions.

### 3.5 Method Validation Protocol

#### 3.5.1 Selectivity and Specificity

The proposed LC method was developed to guarantee sufficient selectivity by encouraging peak purity data and mass characterization by LC-MS-IT-TOF analysis. The peak purity observed under oxidative degradation conditions was 0.9998; under alkaline degradation conditions, 0.9995; and under acidic degradation conditions, 0.9997. Also, blank chromatograms and spectra for human plasma and urine samples were examined, and any interference was observed with the analyte peak. The obtained overlay PDA and total ion chromatograms (TICs) are given in Figures S9 and S10 for spiked and blank human urine and plasma samples, respectively.

#### 3.5.2 Linearity and Range

For each calibration solution, at least three injections were utilized, and average signal intensities were calculated. Results were interpreted using a linear regression model. For the calculation, the ratio of the concentration to the peak area was used. The linearity ranges were 1.4–55.84 and 2.8–11.7 μg/mL for LC-MS/MS and LC-PDA, respectively. All linearity data is given in Table 1. The linearity coefficient was calculated to be 0.999. Furthermore, the results of intraday and interday linearity indicate no significant difference in the data. The analysis-of-variance study using one-way ANOVA resulted in a *p* value greater than 0.05.

#### 3.5.3 Precision

**Figure 4:**
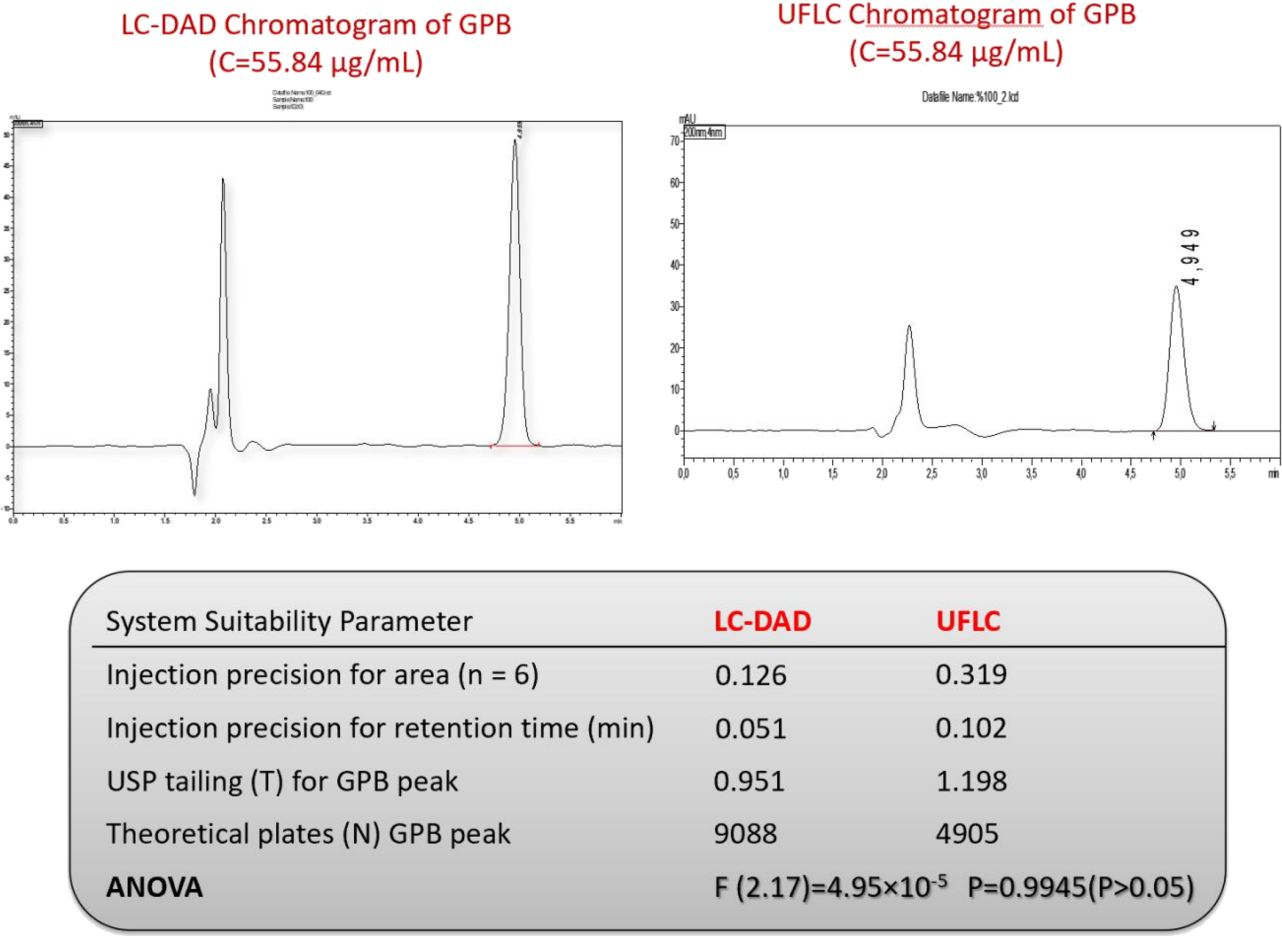
Interlaboratory studies of the LC-PDA method of GPB.

The calculated precision data for the developed method are available in Table 3. Additionally, we conducted and evaluated an interlaboratory comparison using one-way analysis of variance, and Figure 4 presents the obtained data. The results are consistent, indicating that the interlaboratory evaluation was adequate. The optimized method conditions in HPLC were accurately adapted for use in a UPLC-PDA system for encouraging interlaboratory comparisons. In the study, the linearity equation was obtained as *y* = 6808.7*x* – 7234.5 and *R*^2^ was obtained as 0.9982 with perfect linearity. A similar study could not be done for the LC-MS/MS method due to the lack of equipment.

**Table 3:** Recovery Data of GPB for Human Urine and Plasma Samples Using Both LC-MS/MS and LC-PDA Methods

	**urine samples**			**precision**	**accuracy**	
**LC-MS/MS**	**added (μg/mL)**	**found (μg/mL)**	**SD**	**RSD (%)**	**recovery (%)**	**error (%)**
	22.336	22.335	0.227	1.01	99.99	–0.01
	27.920	28.102	0.022	0.083	100.7	+0.70
	33.504	33.088 ±	0.087	0.262	98.76	–1.24
	**plasma samples**			**precision**	**accuracy**	
	**added (μg/mL)**	**found (μg/mL)**	**SD**	**RSD (%)**	**recovery (%)**	**error (%)**
	22.336	22.359	0.084	0.376	100.1	+0.11
	27.920	27.924	0.145	0.519	100.0	+0.01
	33.504	32.902	0.197	0.598	98.20	–1.80

	**urine samples**			**precision**	**accuracy**	
**LC-PDA**	**added (μg/mL)**	**found (μg/mL)**	**SD**	**RSD (%)**	**recovery (%)**	**error (%)**
	44.672	42.335	0.216	0.511	98.69	–1.03
	55.840	53.856	0.399	0.740	96.45	–3.55
	67.008	66.317	0.348	0.525	94.77	–5.23
	**plasma samples**			**precision**	**accuracy**	
	**added (μg/mL)**	**found (μg/mL)**	**SD**	**RSD (%)**	**recovery (%)**	**error (%)**
	44.672	42.114	0.128	0.304	94.27	+1.8
	55.840	53.657	0.162	0.301	96.09	–3.9
	67.008	68.193	0.440	0.644	101.8	–5.73

#### 3.5.4 Accuracy

The accuracy of the proposed method was evaluated in different complex matrix mediums, such as plasma and urine. The authors did recovery studies with different matrices to show that the new method is not affected by the parts of the matrix. According to the accuracy results, it can be said that the methods have sufficient accuracy in a complex matrix. Its pharmaceutical formulation Ravicti (Horizon Therapeutics) is not sold in Turkey, yet. Ravicti has 1.1 g/mL GPB, which corresponds to a density of 1.1 g/mL. It does not include any excipient, so pseudo formulation could not be prepared for recovery experiments.

#### 3.5.5 Limit of Detection and Quantification

Ratios of signal-to-noise (S/N) were used to figure out the method’s limits of detection (LOD) and quantification (LOQ). Solutions were prepared corresponding to S/N values of 3 and 10. Furthermore, these solutions are executed three times. These methods exhibit low LOD and LOQ concentrations. For the LC-MS/MS and LC-PDA methods, the calculated S/N values for LOD and LOQ were 3.91, 3.58, 10.22, and 9.14, respectively. Figure S11 also provides the TIC and PDA chromatograms for each demonstrative LOD and LOQ value. LOD and LOQ values are 0.105 and 1.149 ng/mL for the LC-MS/MS method, respectively, and 0.69 μg/mL and 0.96 μg/mL for the LC-PDA method.

#### 3.5.6 Stability

Stability experiments have been performed for GPB. Analyses were conducted three times: at 24 and 48 h under room conditions to assess short-term stability, 3 weeks at −20 °C for long-term stability, and following three freeze–thaw cycles. The stability results indicate their recovery at the RSD% and 95% confidence level, as shown in Table 4.

**Table 4:** Stability of Standard Solutions for the GPB (55.84 μg/mL)

short-term stability (24 h room temperature)		short-term stability (48 h room temperature)		long-term stability (3 weeks, –20 °C)		freeze–thaw stability (3 cycles)	
found (mean ± CI^a^)	difference (%)	found (mean ± CI^a^)	difference (%)	found (mean ± CI^a^)	difference (%)	found (mean ± CI^a^)	difference (%)
54.326 ± 0.20	–2.71	53.25 ± 0.41	–4.64	54.263 ± 0.42	–2.823	57.262 ± 0.50	+2.55

a 95% confidence interval.

#### 3.5.7 Robustness

During the optimization stage, we determined which parameters alter the response the most. Moreover, according to these parameters, a robustness study was designed. These parameters are the flow rate, percentage of the mobile phase organic component, column temperature, and mobile phase additive amount. As a result of the calculations, it was seen that the change in the method parameters of ±10% was less than 5% in the height equivalent to a theoretical plate, peak area, and retention time from the SST (the results are not presented here but can be presented upon request). The overall robustness calculation of LC method for each detector is given in Figure S12.

### 3.6 Whiteness Evaluation of the Current Method

The sustainability approach has gained popularity over the last 30 years in both industrial and research areas in chemistry. In 1990, GAC principles initiated this approach, which has since expanded its scope. The algorithms such as AGREE, ecoscale, NEMI, and GAPI mostly focus on green, environmentally friendly approaches. Although these evaluations are beneficial, they may also possess certain drawbacks.

WAC is a newly proposed approach by Nowak et al. that utilizes RGB 12 algorithms. The calculation sheets are available as MS Excel files, which serve as a supplementary file for the software product of the calculation.^18^ WAC is a part of GAC, but its scope is more detailed. WAC evaluates various stages of the developed method. WAC includes 12 principles similar to GAC, but these principles assess not only the greenness of the proposed methods but also their applicability and practicality. The WAC categorizes each principle via a color code, and each color code evaluates different perspectives of the proposed method. The GAC is explained with a green color code, and it evaluates green titles: four different titles—G1, toxicity of reagents; G2, amount of waste; G3, energy consumption; G4, impact on natural life.

The main additions make it different from the GAC evaluation tools. In the red and blue parts, there are also four separate sections. Red refers to the validation criteria: R1, scope of the method; R2, quantification and detection limits; R3, precision; R4, accuracy. Blue parts interpret the economic criteria: B1, cost; B2, time; B3, requirements; B4, operational simplicity. The method for scoring is extremely simple and involves using an Excel sheet. Suitable scores must be written in the gray column; 0 is the worst result, and 100 is well. The written scores enable development of the final scores for the method. Moreover, this final score relates to the whiteness of the method. Each score contributes to the color density of the method; e.g., a perfect method is shown as 100% white, which means each section, red, green, and blue was scored 100 and completed as red, green, and blue, respectively.

When the RGB12 scores were examined, for the red section, both methods were acceptable. The HPLC-PDA method had a higher score on the R1 part than the LC-MS-MS method because it looked at degradation products. On the other hand, the LC-MS/MS method has lower LOD and LOQ values, so it obtained a higher score on the R2 part. The green part was nearly scored similarly for both methods since the common use of solvents and reagents (LC-MS/MS) was lowly scored as consuming energy sources.

**Figure 5:**
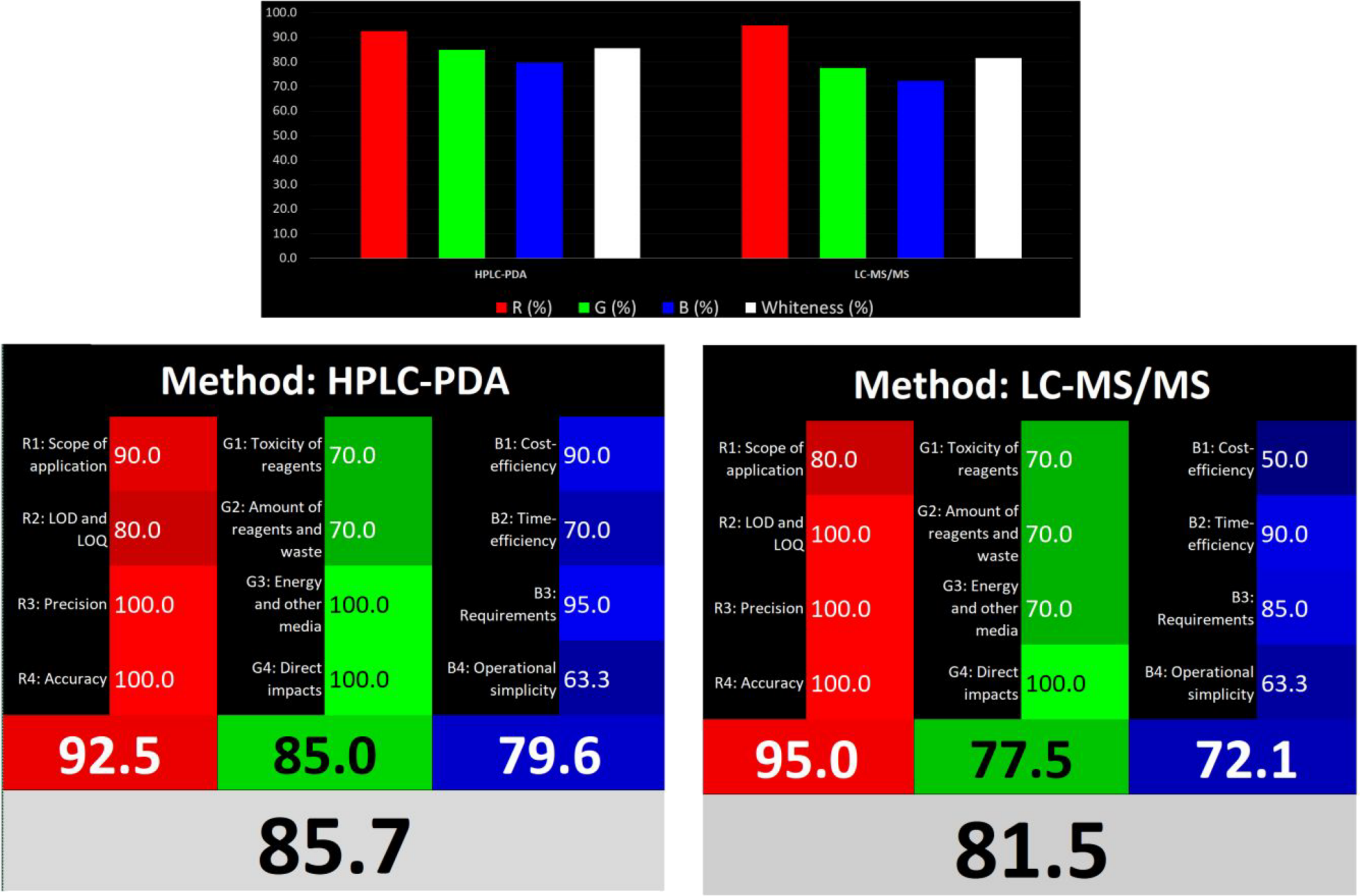
WAC scores for the HPLC-PDA and LC-MS/MS methods for the GPB.

From a practical perspective, HPLC-PDA offers cost advantages, whereas LC-MS/MS delivers a time efficiency. Due to its more intricate optimization procedure, LC-MS/MS is considered more advanced in instrument skills compared to HPLC-PDA, resulting in a lower score. Both systems underwent a comparable assessment of operational simplicity, yielding identical scores. The total scores for the HPLC-PDA and LC-MS/MS were 85.7 and 81.5, respectively, as shown in Figure 5.

### 3.7 Comparisons of LC-PDA and LC-MS/MS

To conclude, the LC-MS/MS and LC-PDA methods have their own advantages when compared. Regarding disadvantages, we observed no deficiency, deadlock, or inadequacy. The LC-PDA method has classical advantages that are known in the literature. It is an analytical instrument that is found in almost every drug analysis laboratory. The LC-MS/MS method requires a higher purity consumption and is an expensive system. It was found that both methods gave similar results for precision, repeatability, and robustness of SSTs during the tests. It is also easy to use either method. However, the LC-MS/MS method could reach approximately 1000 times lower concentrations with LOQ and LOD concentrations and a linear range. These ranges are not necessary for bulk and pharmaceutical formulation analyses and can even be considered a loss in terms of workload and solution dilution. On the other hand, it provides the necessary LOD and LOQ for the analysis of GPB in serum and plasma samples. The analytical method is suitable for the purpose and can meet needs by developing it by considering these benefits and impossibilities.

## 4 Conclusions

In summary, the present paper presents one set of chromatographic conditions applicable to three distinct techniques: LC-PDA, LC-MS/MS, and LC-MS-IT-TOF. The initial stability-indicating liquid chromatography method(s) suggested for quantifying GPB alongside its degradation product. The initial liquid chromatography method(s) was suitable for the analysis of both urine and plasma samples. A new degradation product was found. The identical LC approach is applicable to other detectors. The technique was evaluated by using urine and plasma specimens. The transferability of the method was examined and achieved. Analysis of Ravicti is unfeasible due to procurement issues. Ravicti contains no excipients; hence, a pseudo formulation could not be developed for recovery trials. In addition, the efficiency of the developed methods was evaluated in detail with a whiteness measurement tool. With all of the data it contains, the article in question has the quality and scope to fill an important gap for the prodrug GPB in pharmacokinetic and quality control studies.

This study developed and successfully implemented a method for use with three different analytical instruments: LC-MS/MS, LC-PDA, and LC-MS-IT-TOF. The method achieved LOQ values of 1.15 ng/mL for the MS detector and 0.96 μg/mL for the PDA detector. The method’s adaptability was shown by successfully using it on a different HPLC system, which allowed the accurate and precise detection of the GPB compound in both plasma and urine. Additionally, high-resolution mass analysis identified a novel degradation product. Using whiteness assessments, we systematically evaluated the method’s environmental compatibility, highlighting its analytical performance in terms of practical and green analytical attributes.

## Data Availability

The majority of the data used to support the findings of this study are included within the article. Other data are available from the corresponding author upon request.

## Supporting Information Available

Forced degradation conditions of GPB, tested stationary phases for separation of GPB in LC-MS/MS, possible fragmentation mechanism of GPB in LC-MS/MS, overlay PDA chromatogram of forced degradation conditions for blank and GPB solutions, robustness of the current liquid chromatographic method, and mass balance of GPB after 18 h forced degradation conditions (PDF)

## References

[ref1] Waisbren S. E.; Cuthbertson D.; Burgard P.; Holbert A.; McCarter R.; Cederbaum S.; Biochemical markers and neuropsychological functioning in distal urea cycle disorders. J. Inherited Metab. Dis. 2018, 41 (4), 657–667. 10.1007/s10545-017-0132-5. 29423830 PMC6041144

[ref2] Stepien K. M.; Geberhiwot T.; Hendriksz C. J.; Treacy E. P. Challenges in diagnosing and managing adult patients with urea cycle disorders. J. Inherited Metab. Dis. 2019, 1136 10.1002/jimd.12096. 30932189

[ref3] Stone W. L.; Jaishankar G. B., Urea Cycle Disorders. In StatPearls [Internet]; StatPearls Publishing: 2019. 29493985

[ref4] Berry S. A.; Longo N.; Diaz G. A.; McCandless S. E.; Smith W. E.; Harding C. O.; Zori R.; Ficicioglu C.; Lichter-Konecki U.; Robinson B.; Vockley J. Safety and efficacy of glycerol phenylbutyrate for management of urea cycle disorders in patients aged 2 months to 2 years. Mol. Genet. Metab. 2017, 122 (3), 46–53. 10.1016/j.ymgme.2017.09.002. 28916119

[ref5] McGuire B. M.; Zupanets I. A.; Lowe M. E.; Xiao X.; Syplyviy V. A.; Monteleone J.; Gargosky S.; Dickinson K.; Martinez A.; Mokhtarani M.; Scharschmidt B. F. Pharmacology and safety of glycerol phenylbutyrate in healthy adults and adults with cirrhosis. Hepatology 2010, 51 (6), 2077–2085. 10.1002/hep.23589. 20512995 PMC3733097

[ref6] Oishi K.; Diaz G. A. Glycerol phenylbutyrate for the chronic management of urea cycle disorders. Expert Review of Endocrinology & Metabolism 2014, 9 (5), 427–434. 10.1586/17446651.2014.945908. 30736206

[ref7] Berry S. A.; Lichter-Konecki U.; Diaz G. A.; McCandless S. E.; Rhead W.; Smith W.; LeMons C.; Nagamani S. C. S.; Coakley D. F.; Mokhtarani M.; Scharschmidt B. F.; Lee B. Glycerol phenylbutyrate treatment in children with urea cycle disorders: pooled analysis of short and long-term ammonia control and outcomes. Mol. Genet. Metab. 2014, 112 (1), 17–24. 10.1016/j.ymgme.2014.02.007. 24630270 PMC4382922

[ref8] Optimize RAVICTI® (glycerol phenylbutyrate) Oral Liquid Dose. https://www.ravictihcp.com/-/media/Themes/Horizon/Ravictihcp/Ravictihcp/Documents/RAVICTI-Dose-Considerations.pdf (accessed 10.02.2025).

[ref9] Ott P.; Eriksen P. L.; Kjærgaard K.; So̷rensen M.; Thomsen K. L.; Vilstrup H. Down the road towards hepatic encephalopathy. The elusive ammonia–what determines the arterial concentration?. Metab. Brain Dis. 2024, 40 (1), 1–13. 10.1007/s11011-024-01435-3. 39621139 PMC11611965

[ref10] Search Orphan Drug Designations and Approvals. U.S. Food and Drug Administration https://www.accessdata.fda.gov/scripts/opdlisting/oopd/detailedIndex.cfm?cfgridkey=203505 (accessed 10.02.2025).

[ref11] ICH Q1A (R2) Stability testing of new drug substances and drug products - Scientific guideline. European Medicines Agency https://www.ema.europa.eu/en/ich-q1a-r2-stability-testing-new-drug-substances-drug-products-scientific-guideline.

[ref12] Özcan S.; Erdoğan Uzunoğlu Ü.; Levent S.; Can N. Ö. Liquid chromatographic determination of lumacaftor in the presence of ivacaftor and identification of five novel degradation products using high-performance liquid chromatography ion trap time-of-flight mass spectrometry. J. Sep. Sci. 2023, 46 (17), 2300228 10.1002/jssc.202300228. 37409384

[ref13] Shaikh T. Impurities characterization in pharmaceuticals: A review. Available at SSRN 3958603, 2019.

[ref14] Impurities in new drug products Q3B(R2). Curr. Step 2006, 4, 1–5.

[ref15] Géhin C.; Holman S. W. Advances in high-resolution mass spectrometry applied to pharmaceuticals in 2020: a whole new age of information. Analytical Science Advances 2021, 2 (3–4), 142–156. 10.1002/ansa.202000149. 38716455 PMC10989654

[ref16] Deschamps E.; Calabrese V.; Schmitz I.; Hubert-Roux M.; Castagnos D.; Afonso C. Advances in ultra-high-resolution mass spectrometry for pharmaceutical analysis. Molecules 2023, 28 (5), 2061 10.3390/molecules28052061. 36903305 PMC10003995

[ref17] Vemuri D. K.; Akshinthala P.; Konduru N.; Kowtharapu L. P.; Katari N. K.; Jonnalagadda S. B.; Gundla R. Unique quality by design approach for developing HPLC and LC-MS method for estimation of process and degradation impurities in pibrentasvir, antiviral agent for hepatitis C. ACS omega 2022, 7 (51), 47650–47661. 10.1021/acsomega.2c04617. 36591161 PMC9798757

[ref18] Nowak P. M.; Wietecha-Posłuszny R.; Pawliszyn J. White analytical chemistry: an approach to reconcile the principles of green analytical chemistry and functionality. TrAC Trends in Analytical Chemistry 2021, 138, 116223 10.1016/j.trac.2021.116223.

