# Supplementary material for: LC-MS/MS and LC-PDA Methods for Robust Determination of Glycerol Phenylbutyrate in Biological Fluids and High-Resolution Mass Spectrometric Identification of Forced Degradation Product and Its Whiteness
Source: ACS Omega. 2025 Apr 25;10(17):17836–46. doi: 10.1021/acsomega.5c00569 (PMC12060053; doi:10.1021/acsomega.5c00569)
Supplement: Supplementary file 1 — ao5c00569_si_001.pdf [file ao5c00569_si_001.pdf]

SUPPLEMENTARY MATERIAL

LC-MS/MS and LC-PDA Methods for Robust Determination of Glycerol Phenylbutyrate in Biological Fluids and High-Resolution Mass Spectrometric Identification of Forced Degradation Product and its whiteness

Serkan Levent[1,2*], Abeer Elriş[1], Hazal Avcı[1,2], Ülfet Erdoğan Uzunoğlu[3], Saniye Özcan[1,2], Nafiz Öncü Can[1,2]

Authors Affiliations:

[1]Department of Analytical Chemistry, Faculty of Pharmacy, Anadolu University, 26470, Eskisehir, Turkey.

[2]Central Analysis Laboratory (MERLAB), Faculty of Pharmacy, Anadolu University, 26470 Eskisehir, Turkey.

E-mail addresses and ORCID numbers:

Serkan Levent: serkanlevent@anadolu.edu.tr	0000-0003-3692-163X
Abeer Elriş: aalreesh@anadolu.edu.tr	0000-0001-8467-478X
Hazal Avcı: hazal_avci@anadolu.edu.tr	0009-0000-8479-3615
Ülfet Erdoğan Uzunoğlu: erdogau@clarkson.edu	0000-0001-6189-0818
Saniye Özcan: saniyeozcan@anadolu.edu.tr	0000-0002-5492-0457
Nafiz Öncü Can: nafizoc@anadolu.edu.tr	0000-0003-0280-518X

[*]To whom correspondence should be addressed:

Department of Analytical Chemistry,
Faculty of Pharmacy, Anadolu University,
Yunusemre Campus, Eskisehir 26470, Turkey
Tel: +90 222 3350750 ext. 3619 Fax: +90 222 3350750
e-mail: serkanlevent@anadolu.edu.tr

- **Degradation Conditions:**
  - 1 mg/mL GPB in 0.5 N HCl & Blank (60 ºC, 1 hour)
  - 1 mg/mL GPB in 0.5 N NaOH & Blank (60 ºC, 1 hour)
  - 1 mg/mL GPB in 3% H_2O_2 & Blank (60 ºC, 1 hour)
  - 1 mg/mL GPB in UV light & Blank (60 ºC, 1 hour)
  - 1 mg/mL GPB & Blank (60 ºC, 1 hour)

Figure S1. Forced degradation conditions of GPB.

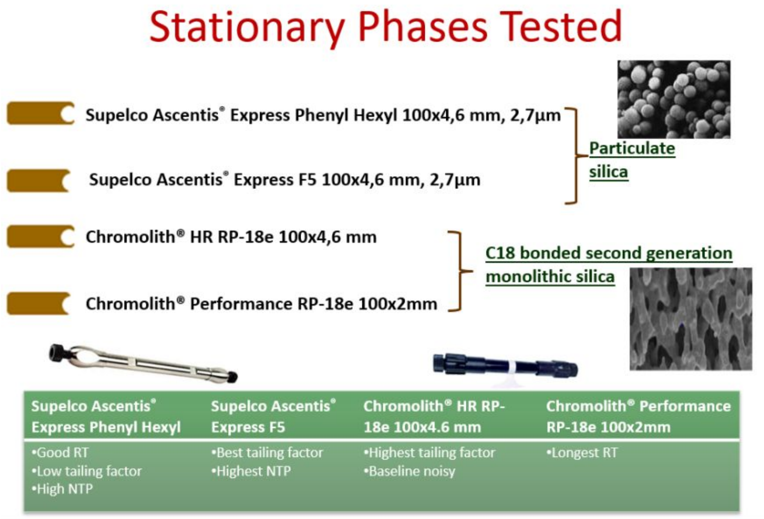

Figure S2. Tested stationary phases for separation of GPB in LC-MS/MS.

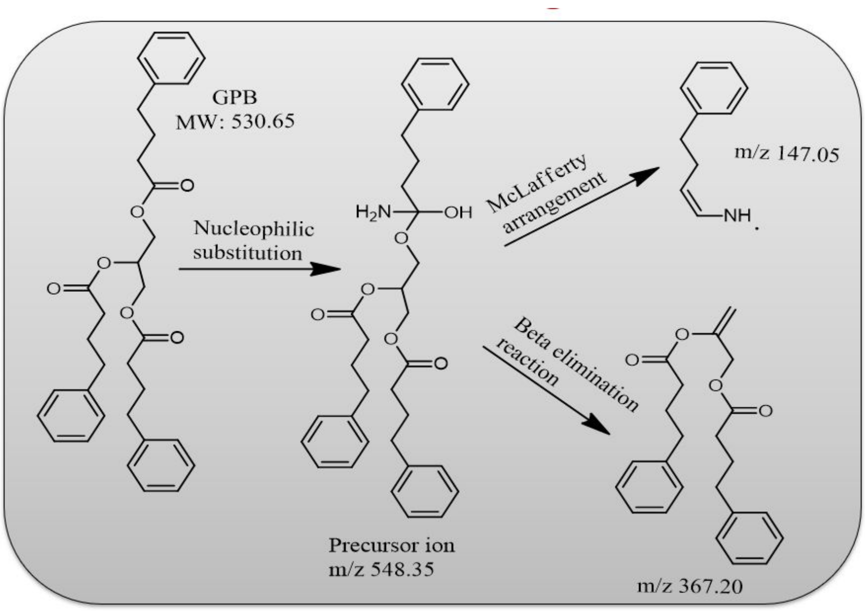

Figure S3. Possible fragmentation mechanism of GPB in LC-MS/MS.

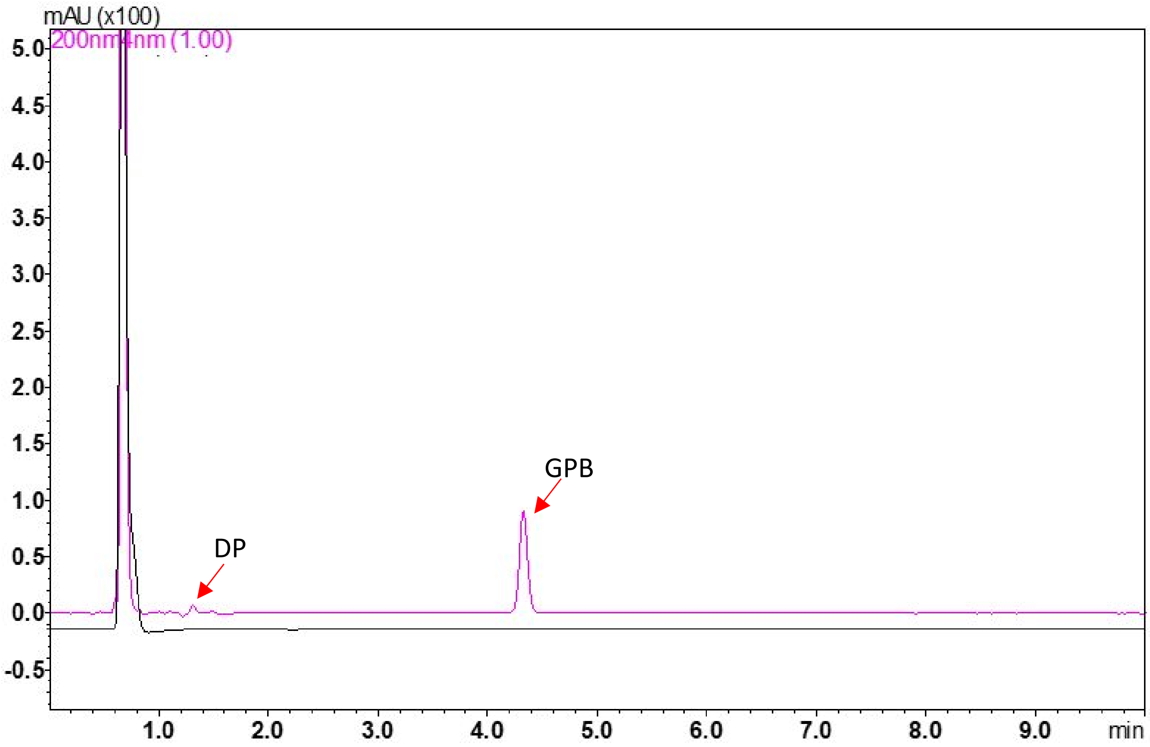

Figure S4. The overlay PDA chromatogram oxidative forced degradation conditions for blank and GPB solutions.

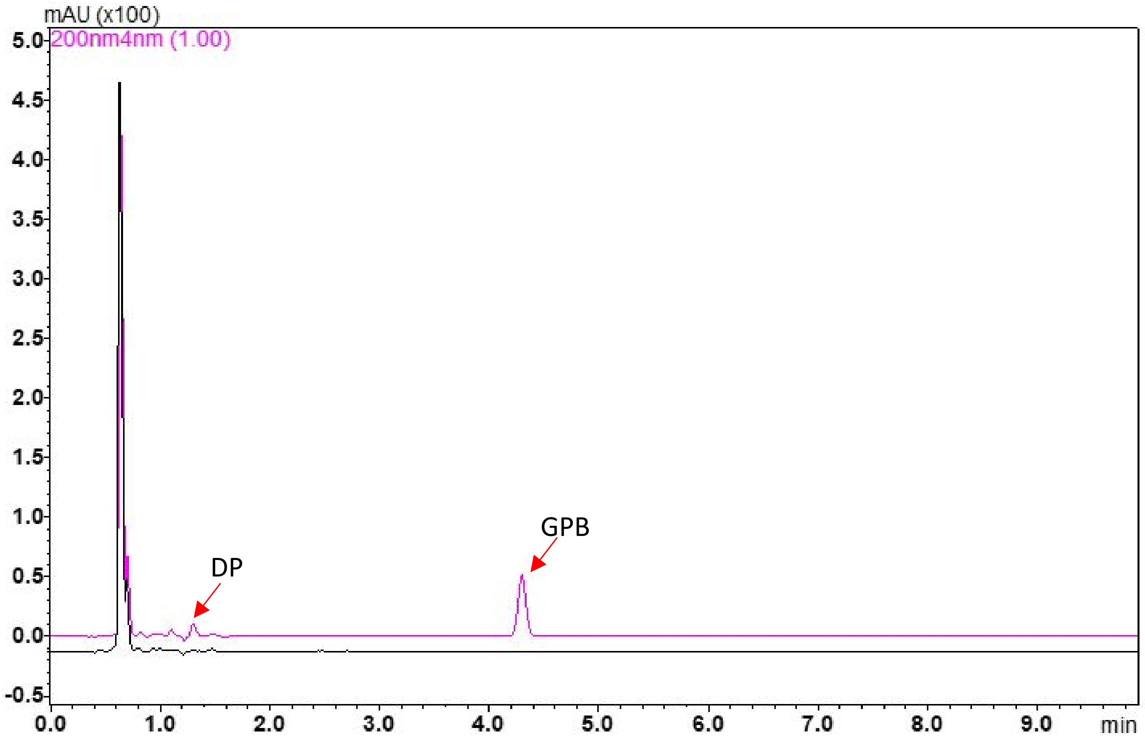

Figure S5. The overlay PDA chromatogram acid forced degradation conditions for blank and GPB solutions.

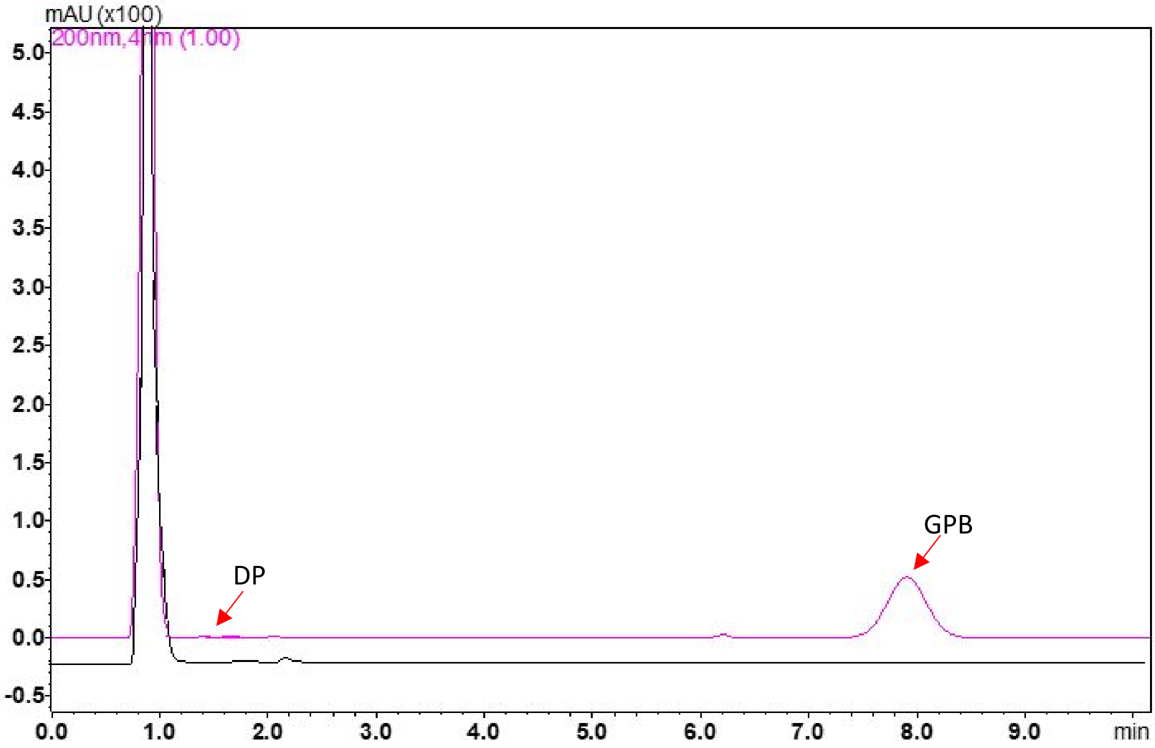

Figure S6. The overlay PDA chromatogram basic forced degradation conditions for blank and GPB solutions.

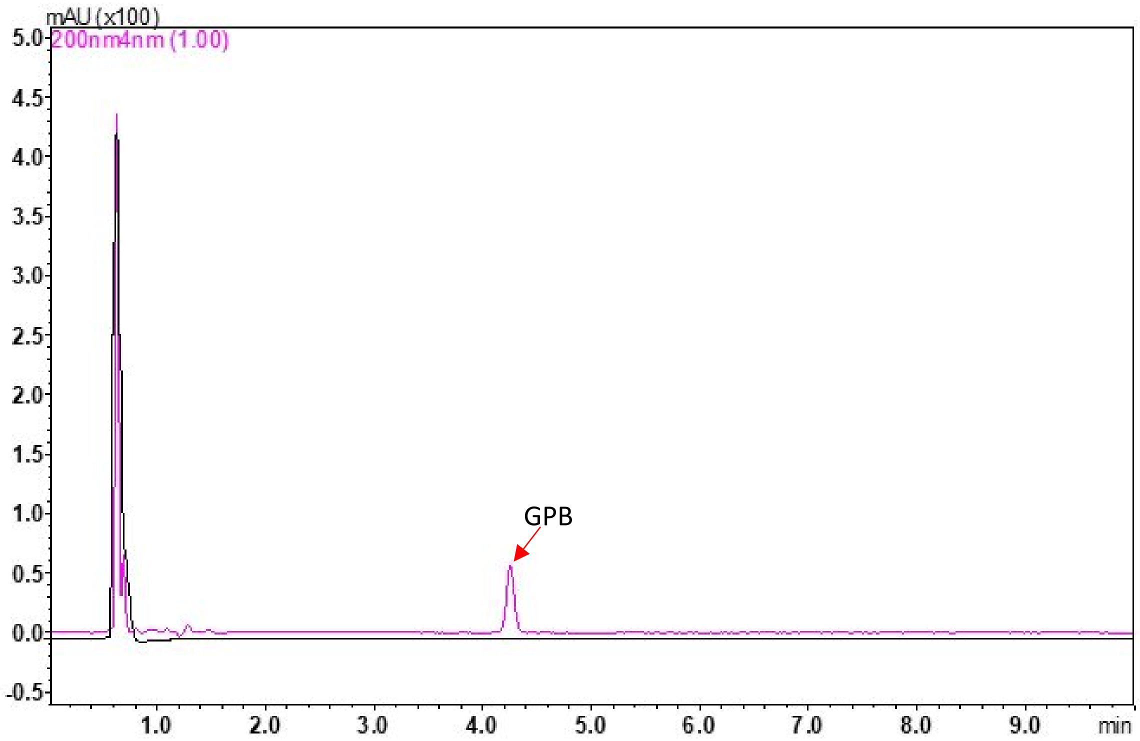

Figure S7. The overlay PDA chromatogram heat forced degradation conditions for blank and GPB solutions.

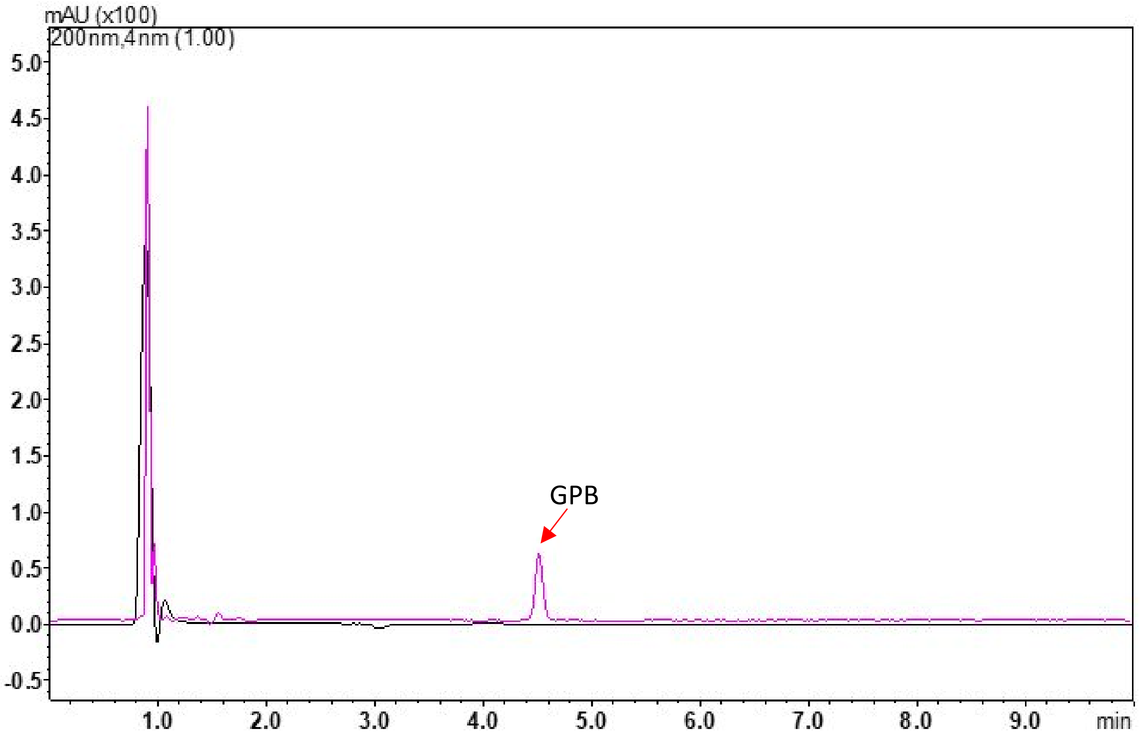

Figure S8. The overlay PDA chromatogram UV light forced degradation conditions for blank and GPB solutions.

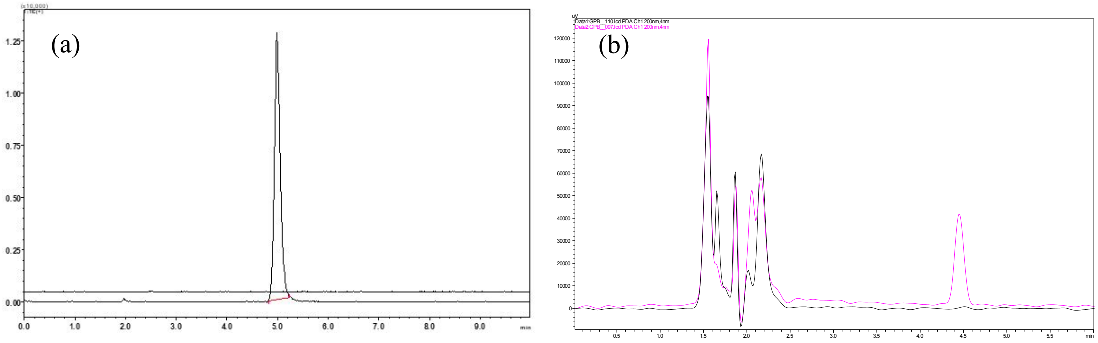

Figure S9. Overlay TIC spectrums (a) and PDA chromatogram (b) of spiked human urine (100% concentration) and blank human urine samples.

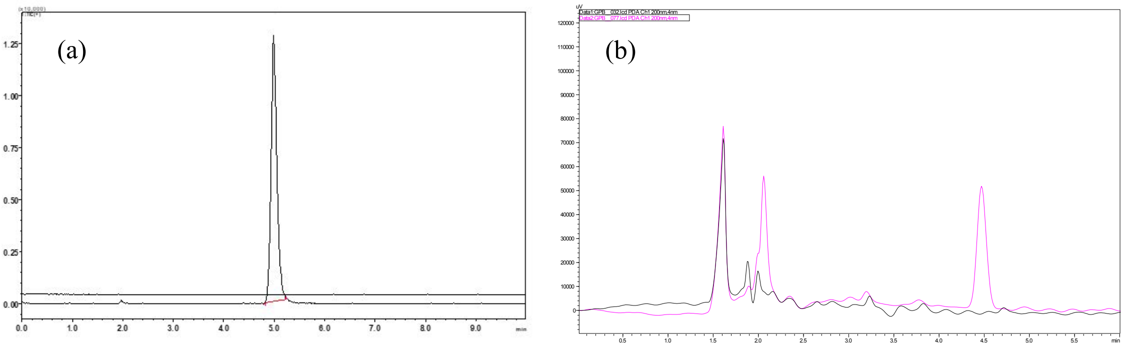

Figure S10. Overlay TIC spectrums (a) and PDA chromatogram (b) of spiked human plasma (100% concentration) and blank human plasma samples.

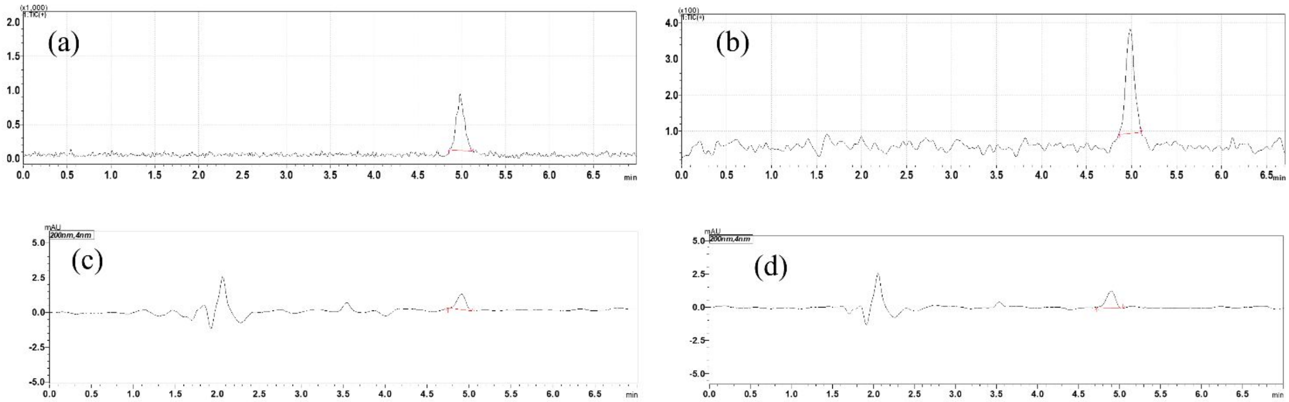

Figure S11. The obtained TIC and PDA chromatograms of LOD (a, c) and LOQ (b, d), respectively.

Robustness of LC-PDA System

Robustness Of Column				Robustness Of Mobile Phase			
Column Temp.	RT (min)	RT RSD (%)	Difference (%)	B (%)	RT (min)	RT RSD (%)	Difference (%)
**36 C°**	5.011	0.070	+2.321	**79**	3.898	0.128	-20.40
**40 C°**	4.892	0.051		**75**	4.892	0.051	
**44 C°**	4.946	0.160	+1.001	**71**	6.641	0.061	+35.63

Robustness Of Flow Rate				Robustness Of Wavelength			
FR (mL/min)	RT (min)	RT RSD (%)	Difference (%)	±4 nm	RT (min)	RT RSD (%)	Difference (%)
0.45	5.481	0.100	+11.92	**196**	4.963	0.031	+1.355
0.50	4.892	0.051	5.271	**190**	4.892	0.051	
0.55	4.528	0.285	-7.528	**204**	4.964	0.051	+1.382

Robustness Of Wavelength

	Peak area	%Difference	Peak Height	%Difference
204 nm	676987.3	85.9	92820.3	86.2
200 nm	364185	-	49844.3	-
196 nm	520586.2	85.9	71332.3	86.2

Robustness of LC-MS/MS System

Robustness Of Column				Robustness Of Mobile Phase				Robustness Of Flow Rate			
Column Temp.	RT (min)	RT RSD (%)	Difference (%)	B (%)	RT (min)	RT RSD (%)	Difference (%)	FR (mL/min)	RT (min)	RT RSD (%)	Difference (%)
**36 C°**	5.121	0.147	-2.839	**79**	3.983	0.276	-24.429	0.45	5.55	0.160	5.387
**40 C°**	5.271	0.190		**75**	5.271	0.190		0.50	5.271	0.190	
**44 C°**	5.060	0.149	-4.003	**71**	6.789	0.100	28.812	0.55	4.604	0.151	0.163

Figure S12. Robustness of the current liquid chromatographic method.

Table S1. Mass balance of GPB after 18 hours forced degradation conditions.

Stress Condition	Assay of GPB	Total Impurities	Mass Balance (Assay + Total Impurities)
**Thermal Acidic Degradation**	98.5 %	1.4 %	99.9 %
**Thermal Basic Degradation**	99.5 %	0.3 %	99.8 %
**Thermal Oxidative Degradation**	96.1 %	3.9 %	100.0 %
**Photolytic Degradation**	99.9 %	n.d.	99.9 %
**Thermal Degradation**	99.9 %	n.d.	99.9 %
